# Comparative evaluation of ASA classification and ACE-27 index as morbidity scoring systems in oncosurgeries

**DOI:** 10.4103/0019-5049.65366

**Published:** 2010

**Authors:** Mary Thomas, Nebu Abraham George, Balagopal Prabhakar Gowri, Preethi Sara George, Paul Sebastian

**Affiliations:** Department of Anaesthesiology, Regional Cancer Centre, Thiruvananthapuram, Kerala, India; 1 Department of Surgical Oncology, Regional Cancer Centre, Thiruvananthapuram, Kerala, India; 2 Department of Biostatistics, Regional Cancer Centre, Thiruvananthapuram, Kerala, India

**Keywords:** Morbidity prediction, perioperative complications, surgery

## Abstract

The primary intention of the study was to find out whether Adult Comorbidity Evaluation Index (ACE-27) was better than the American Society of Anaesthesiologists’ (ASA) risk classification system in predicting postoperative morbidity in head and neck oncosurgery. Another goal was to identify other risk factors for complications which are not included in these indexes. Univariate and multivariate analyses were performed on 250 patients to determine the impact of seven variables on morbidity-ACE-27 grade, ASA class, age, sex, duration of anaesthesia, chemotherapy and radiotherapy. In univariate analysis ACE-27 index, ASA score, duration of anaesthesia, radiotherapy and chemotherapy were significant. As both comorbidity scales were significant in univariate analysis they were analyzed together and separately in multivariate analysis to illustrate their individual strength. In the first multivariate analysis (excluding ACE-27 grade) ASA class, duration of anaesthesia, radiotherapy and chemotherapy were significant. The positive predictive value (PPV) of this model to predict morbidity was 60.86% and negative predictive value (NPV) was 77.9%. The sensitivity was 75% and specificity 62.2%. In the second multivariate analysis (excluding ASA class) ACE-27 grade, duration of anaesthesia and radiotherapy were significant. The PPV of this model to predict morbidity was 62.1% and NPV was 76.5%. The sensitivity was 61.6% and specificity 70.9%. In the third multivariate analysis which included both ACE-27 grade and ASA class only ASA class, duration of anaesthesia, radiotherapy and chemotherapy remained significant. In conclusion, ACE-27 grade and ASA class were reliable predictors of major complications but ASA class had more impact on complications than ACE-27 grade.

## INTRODUCTION

The anaesthesiologist is the perioperative medical specialist and the only preoperative evaluation physician who can truly evaluate the risks associated with anaesthesia, discuss these risks with the patient, and manage them intraoperatively. Anaesthesia care is no longer limited to the operating room. Many departments of anaesthesiology have even changed their official departmental titles to include anaesthesia and “perioperative care.”[1]

Head and neck cancer has a higher incidence in people older than 50 years, primarily because of its relationship with chronic tobacco and alcohol exposure[2] Besides cancer, these exposures are also associated with other significant systemic comorbidities such as pulmonary, cardiovascular, hepatic and metabolic diseases, which modify treatment tolerance and influence short-term prognosis.[3] However, there is a general paucity of literature regarding the prevalence of comorbid conditions and its impact on perioperative anaesthetic outcome in patients undergoing major cancer surgery.[4]

Various instruments have been developed to assess the effect of comorbidities on patient survival.[5] We chose Adult Comorbidity Evaluation Index (ACE-27) as it has been widely validated for head and neck cancers.[6] Piccirillo developed the ACE-27 Index.[6] It is a modification of the Kaplan-Feinstein Index.[7] It gives us a comprehensive review of all systems.[8] The American Society of Anaesthesiologists’ (ASA) risk classification system is actually an index for perioperative risk, but it can also be used to evaluate comorbidity because it describes a patient’s physical status prior to surgery.[8] The current ASA classification was developed in 1941 by Meyer Saklad.[9]

This primary intention of the study was to find out whether ACE-27 was better than the commonly used ASA system in predicting perioperative complications in head and neck oncosurgery. We also studied the effect of other probable risk factors for these patients which are not included in these indexes like age, sex, preoperative chemotherapy, radiation therapy and duration of anaesthesia.

## METHODS

After obtaining clearance from the Institutional Review Board data were collected prospectively using a computer database developed for the head and neck surgery unit under the Regional Cancer Centre, Thiruvananthapuram, a tertiary referral institution providing hospitalized care in South India. Informed consent for this study was not required as all patients registered for treatment in our institution sign a consent form allowing clinical data to be taken for research purposes. The study set included 250 patients treated surgically for head and neck cancer between January 2007 and January 2009.

The following criteria were used for inclusion in the study: a histologically confirmed diagnosis of squamous cell carcinoma, no distant metastasis, and surgical treatment with a curative purpose, exclusive or as part of a multidisciplinary approach.

The data set included the following patient characteristics: age, sex, ASA class, ACE-27 grade, duration of anaesthesia and previous treatment with chemotherapy or radiation therapy. Outcome measures included the development of complications in the immediate postoperative period (30 days). Major complications were defined according to the system outlined by Farwell *et al*.[10]

The ASA class, assigned by the attending anaesthesiologist was obtained from the original anaesthesia form. Grade 1-A normal healthy patient; Grade 2, 3 and 4, patient with mild systemic disease, severe systemic disease and disease which is a constant threat to life. Grades 5 and 6 are moribund and brain-dead patient respectively. A complete description of the classification is available on http://en.wikipedia.org/wiki/ASA_score.

Patients were also classified according to the ACE-27 data form which includes comorbid conditions of various organ systems such as cardiovascular, respiratory, gastrointestinal, renal, endocrine, neurological, psychiatric, rheumatologic, and immunological systems, as well as malignancy, substance abuse, and body weight. Each category contains three grades (1, mild; 2, moderate; and 3, severe), with the overall comorbidity score defined according to the highest ranked single ailment. Two or more Grade 2 ailments occurring in different organ systems result in a Grade 3 assignment. ACE-27 grades were allotted as 0, 1, 2 or 3 in the hospital information system. A complete ACE-27 data form is available on http://oto.wustl.edu/clinepi/calc.html (Clinical Outcomes Research Office’s Website).

All statistical analyses were performed with SAS software, Version 9.1 (SAS Institute, INC Cary. NC). Medical and surgical postoperative complications were joined into a single variable and used as the outcome measure in all analyses. For determining the impact of predictive factors on morbidity we first performed a univariate analysis with Fisher’s exact test and then a multivariate analysis by logistic regression with odds ratio and 95% confidence intervals. Variables with a level of significance less than or equal to 0.05 in the univariate analysis were included in the multivariate model which was analyzed with a stepwise logistic regression. Interaction effects were sought for all variables included in the model. For the purpose of regression analysis, age and duration of anaesthesia were considered as numerical variables. The other variables were binary. The comorbidity scales were analyzed together as well as separately to illustrate their individual strength.

We generated different probability cut-offs, and tabulated the respective sensitivity, specificity, the positive predictive value (PPV) and negative predictive value (NPV), then decided which was the best cut-off for optimal results. The performance of two tests was compared by plotting the receiver operator characteristic (ROC) curve of each test values. The area under the ROC curve, which ranges from 0 to 1, was used to assess the model discrimination.

## RESULTS

The incidence of postoperative morbidity and mortality was 29.2% (73 patients) and 1.6% (four patients) respectively. Serious medical complications occurred in 36 patients (14.4%) and surgical complications in 37 patients (14.8%). The commonest postoperative surgical problem was flap necrosis (4.4%) and commonest systemic problem was pneumonia (2.4%). The frequencies of postoperative problems are listed in Table 1.

**Table 1:** Major postoperative complications

Postoperative complications		No. (%) of patients with major complications (n=250)
Total serious medical complications	Myocardial ischaemia	2 (0.8%)
	Myocardial infarction	1 (0.4%)
	Congestive failure	1 (0.4%)
	Hypoxia	4 (1.6%)
	Ventilator support >24 h	2 (0.8%)
	Pneumonia	6 (2.4%)
	Adult respiratory distress syndrome	2 (0.8%)
	Bronchospasm	2 (0.8%)
	Other pulmonary complications	1 (0.4%)
	Delirium	1 (0.4%)
Total serious surgical complications	Wound breakdown	8 (3.2%)
	Fistula formation	10 (4%)
	Flap donor and recipient site complications	11 (4.4%)
	Wound hematomas	1 (0.4%)
	Need for additional unexpected procedure	7 (2.8%)
Total serious infections	Surgical site infection deep	2 (1.34)
	Bacteraemia	1 (0.67%)
	Abscess	1 (0.67%)
	Sepsis	1 (0.67%)
Miscellaneous	Renal insufficiency	1 (0.67)
	Alcohol withdrawal	2 (1.34)
	Other miscellaneous	1 (0.67)
	Unexpected transfer	1 (0.67)
Death		4 (1.6%)

The distribution of the various variables of the study set (250 patients) -age, sex, ASA score, ACE-27 grade, duration of anaesthesia and previous treatment (chemotherapy and radiation therapy) is shown in Table 2. The univariate impact of all variables on major complications is detailed in the last column.

**Table 2:** Demographic characteristics of patients in the study set and the Impact of variables on major complications in univariate analysis

Variable	All patients (n =250)	Patients with morbidity (n= 73)	Patients without morbidity (n= 177)	*P* value*
Age in years			
<50	78 (31.2)	23 (29.5)	55 (7.05)	0.994
50-65	123 (49.2)	36 (29.3)	87 (70.7)	
>65	49 (19.6)	14 (28.6)	35 (71.4)	
Sex			
Male	171 (68.4)	54 (73.6)	118 (67.8)	0.447
Female	79 (31.6)	19 (26.4)	59 (32.2)	
American Society of Anaesthesiologists Score (ASA)			
1	89 (35.6)	13 (14.6)	76 (85.4)	0.001*
2	125 (50.0)	45 (36.0)	80 (64.0)	
3	36 (14.4)	15 (41.7)	21 (58.3)	
Adult comorbidity evaluation index-(ACE-27)			
0	67 (26.8)	10 (14)	57 (85.1)	0.003*
1	142 (56.8)	49 (34.5)	93 (65.5)	
2	39 (15.6)	12 (30.8)	27 (69.2)	
3	2 (0.8)	2 (100)	0	
Preoperative chemotherapy			
Yes	49 (19.6)	23 (46.9)	26 (53.1)	0.000*
No	201 (80.4)	50 (24.9)	151 (75.1)	
Preoperative radiotherapy			
Yes	59 (23.6)	31 (52.5)	28 (47.5)	0.000*
No	191 (76.4)	42 (22.0)	149 (78.0)	
Duration of Surgery in minutes			
<180	99 (39.6)	17 (17.2)	82 (82.8)	0.000*
>180	151 (60.4)	56 (37.1)	95 (62.9)	

* *P*<0.05-Significant, Figures in parentheses are in percentage

In univariate analysis neither age nor sex was significantly associated with morbidity. ACE-27 index and ASA score had a statistically significant relationship with postoperative complications, (*P*=0.001) and (*P*=0.003) respectively. Duration of anaesthesia (*P*=0.000), preoperative radiotherapy (*P* = 0.000) and chemotherapy (*P*=0.000) were also significant in predicting postoperative morbidity.

Multivariate logistic regression analysis results are summarized in Tables 3–5. As both ACE-27 grade and ASA class were significant predictors in univariate analysis, three multivariate analyses were performed. The first two multivariate analyses contained all significant variables but only one of the two comorbidity scales (ACE-27 grade or ASA class). The third multivariate analysis contained all significant variables, including both ACE-27 grade and ASA class. The impact on morbidity of all significant variables excluding ACE-27 grade in multivariate (forward selection) analysis is shown in Table 3 and excluding ASA class is shown in Table 4.

**Table 3:** Impact of all significant variables excluding (ACE 27) on morbidity in multivariate (forward selection) analysis

Variable	Odds ratio	95% confidence interval	*P* value
American Society of			
Anaesthesiologists (ASA) score			
ASA(1)	3.969	1.876-8.4	0.001*
ASA(2)	4.351	1.64-11.542	0.003*
Chemotherapy	2.194	1.017-4.730	0.045*
Radiation therapy	2.460	1.223-4.951	0.012*
Duration of anaesthesia	2.772	1.429-5.379	0.003*

* *P*<0.05-Significant

**Table 4:** Impact of all significant variables excluding ASA class on morbidity in multivariate (forward selection) analysis

Variable	Odds ratio	95% confidence interval	*P* value
Adult comorbidity Evaluation index (ACE-27)			
ACE(1)	3.165	1.436-6.974	0.037*
ACE(2)	2.145	0.775-5.936	0.004*
Radiation therapy	3.392	1.762-6.528	0.001*
Duration of anaesthesia	2.585	1.347-4.961	0.004*

* *P*<0.05-Significant

**Table 5:** Impact of all significant variables with ASA class and ACE grade on morbidity in multivariate (forward selection) analysis

Variable	Odds ratio	95% confidence interval	*P* value
American Society of Anaesthesiologists (ASA) score	3.969	1.876-8.4	0.000*
	4.351	1.64-11.542	0.003*
ASA(1)			
ASA(2)			
Chemotherapy	2.194	1.017-4.730	0.045*
Radiation therapy	2.460	1.223-4.951	0.012*
Duration of anaesthesia	2.772	1.429-5.379	0.003*

* *P*<0.05-Significant

In the first multivariate analysis, ASA class, duration of anaesthesia, preoperative radiotherapy and preoperative chemotherapy were significant predictors of morbidity. The odds ratio (OR) and 95% confidence intervals (CIs) associated with them were ASA Class (1) 3.969 (1.876-8.4), ASA Class (2) 4.351 (1.64-11.542), preoperative chemotherapy 2.194 (1.017-4.730), preoperative radiotherapy 2.460 (1.223-4.951) and duration of anaesthesia 2.772 (1.429-5.379) respectively. The overall accuracy of this model (including all the significant variables excluding ACE -27) to predict subjects having morbidity was 75.2%. The positive predictive value (PPV) of this model to predict morbidity was 60.86% and negative predictive value (NPV) was 77.9%. The sensitivity was 75% and the specificity was 62.2%.

In the second multivariate analysis ACE-27 grade, duration of anaesthesia and preoperative radiotherapy were significant. The odds ratio and 95% confidence intervals associated with them were ACE-27 (1) 3.165 (1.436-6.974), ACE-27(2) 2.145 (0.775-5.936), duration of anaesthesia 3.392 (1.762-6.528) and preoperative radiotherapy 2.585 (1.347-4.961) respectively. The overall accuracy of this model (including all the significant variables excluding ASA Class) to predict subjects having morbidity was 72.5%. The PPV of this model to predict morbidity was 62.1% and NPV was 76.5%. The sensitivity was 61.6% and the specificity was 70.9%.

**Figure 1:**
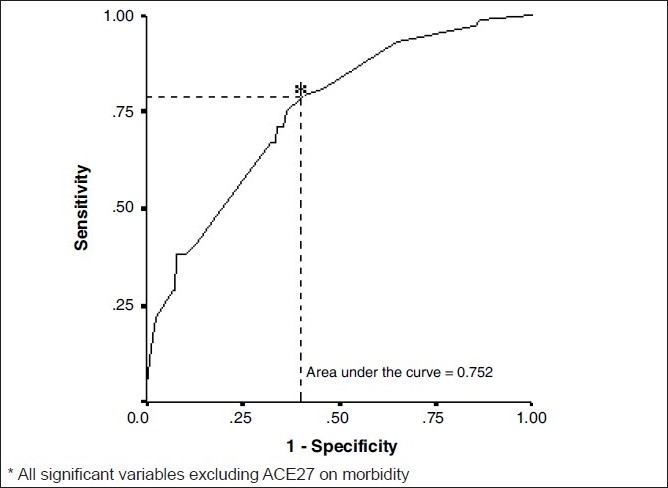
Receiver operating characteristics for Model-I^*^

**Figure 2:**
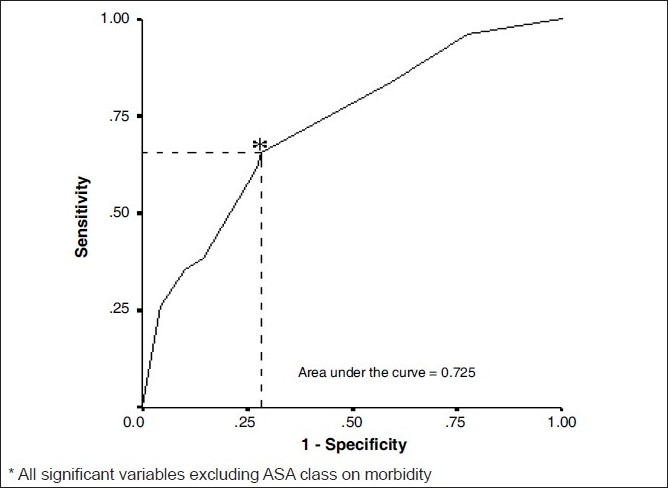
Receiver operating characteristics for Model-II^*^

The area under the Figure 1 ROC curve was 0.752 and Figure 2 ROC curve was 0.725. Figure 1 would be considered to be “good” at separating which one has morbidity from the other without morbidity.

In the third multivariate analysis [Table 5], which included both ACE-27 grade and ASA class, ASA class had more impact on complications than did ACE-27 grade. The ORs (95% CIs) were as follows: ASA Class 1 3.969 (1.876-8.4), ASA Class 2 4.351 (1.64-11.542), preoperative chemotherapy 2.194 (1.017-4.730), preoperative radiotherapy 2.460 (1.223-4.951) and duration of anaesthesia 2.772 (1.429-5.379) respectively. The overall accuracy of this model (including all the significant variables with ACE -27 and ASA class) to predict subjects having morbidity is 75.2%. The PPV of this model to predict morbidity was 60.86% and NPV was 77.9%. The sensitivity was 75% and the specificity was 62.2%.

**Figure 3:**
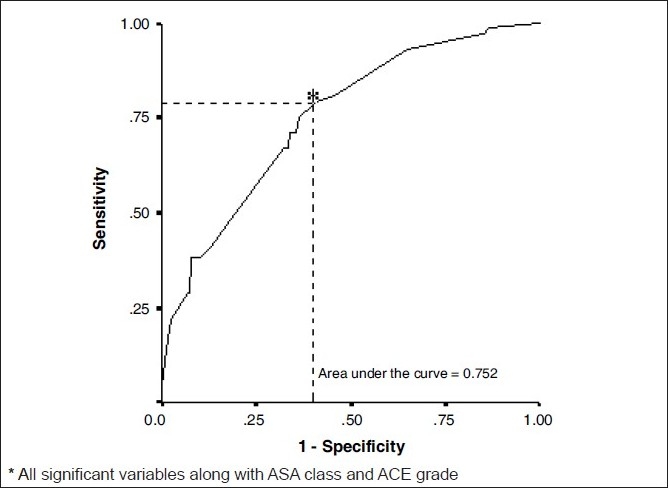
Receiver operating characteristics for Model-III^*^

The area under the Figure 3 ROC curve was 0.752. Figure 3 would be considered to be “good” at separating which one has morbidity from the other without morbidity.

## DISCUSSION

Risk assessment is useful to compare outcomes, control costs, allocate compensation, postpone surgery until interventions improve risk or in assisting in the difficult decision of cancelling or recommending that a procedure not be done when the risks are too high.[1] The ideal risk assessment scoring system should be easy to use, widely applicable, and accurately predict outcome.

The ASA class was a strong predictor of the development of postoperative complications in our study. The correlation between ASA score and postoperative mortality rates has been well established in non-cancer general surgery.[11,12] This scoring system assumes that the age of the patient has no relation to physical fitness, ignores patients with malignancy and surgical complexity.

In head and neck cancer surgery, ASA score had a controversial value. In some previous studies, there was no association between ASA score and postoperative mortality or morbidity.[13–15] On the contrary, others showed a significant correlation between ASA score and postoperative morbidity.[8,16–18] The controversial value achieved with the ASA score is possibly because this system is primarily based on subjective clinical judgments.[19]

Disease-specific comorbidity measures have a conceptual advantage in that specific treatment and outcome issues unique to that population are considered in their development but they may not always perform better than general measures in the prediction of outcome.[20] Outcome indicators for cancer can be evaluated by using various comorbidity indexes like Kaplan-Feinstein Index, Cumulative Illness Rating Scale (CIRS), Charlsons Index, Index of Coexistent Disease (ICED) and Adult Comorbidity Evaluation Index (ACE-27) or modified Kaplan-Feinstein Index. None of these comorbidity indexes were developed on cancer patients but they have been found to be useful in them. Many authors have applied ACE -27 index for describing comorbidity and predicting the overall survival of cancer populations in head and neck cancer and found it reliable.[21,22] A few studies on the Western population have shown that ACE-27 index effectively assesses the probability of postoperative morbidity.[8,23] The incidence of many parameters in the ACE-27 index like peripheral vascular diseases, rheumatologic diseases, obesity, AIDS, hepatic, gastric or pancreatic illness was less than 1% in our study group. Parameters like valvular heart disease and hypothyroidism which are common in our population were not included in the index. A disadvantage of the ACE-27 is that it can be time-consuming.[8]

In our study we found a statistically significant increase in morbidity as duration of anaesthesia increases. Time under general anaesthesia has shown a statistically significant relationship with complication rate and length of hospital stay.[24] In a similar study, Ferrier *et al*., found that anaesthesia time more than eight hours was an independent predictor of complications.[8] Stress of surgery and anaesthesia evoke many metabolic and endocrine changes in otherwise healthy individuals that can lead to postoperative problems.

Patients with a history of cancer may have complications related to disease or treatment.[1] Preoperative radiotherapy and preoperative chemotherapy had a statistically significant increased incidence of complications in multivariate analysis. Akihiro Sakai *et al*., reported an incidence of 27% postoperative complications in post-radiation patients.[25] Since radio-therapy promotes vascular endothelial cell growth leading to fibrosis of the surrounding connective tissue, intubation can become difficult and traumatic as patients can develop trismus and neck stiffness. When surgery is performed on such patients, avascular skin necrosis and fistula formation is more likely to occur. Studies have shown that patients who received preoperative chemotherapy also have an increased incidence of postoperative complications.[26]

Several previous studies found a lower risk of postoperative morbidity in women.[19,27] In our study we found a similar incidence of postoperative complications in both genders like Ferrier *et al*.[8]

A prospective case-control study by Kowalski *et al*., on elderly patients undergoing head and neck surgery failed to identify any increased frequency of postoperative complications or mortality as compared to younger patients.[28] Three other studies had clearly confirmed that age had no significant impact on the incidence of postoperative morbidity.[8,19,29] In our study too age was not associated with increased morbidity.

The burden of comorbidity is clearly a major prognostic factor but the underlying mechanisms are not well understood. It is worthwhile identifying single diseases contributing to the risk of complications so that predisposing factors for post operative morbidity can be avoided. In the meantime, a high comorbidity score should lead to a higher degree of caution by the clinicians. Morbidity scoring systems can be used prospectively to predict morbidity so that patient could be counselled concerning operative risk, to modify surgical procedures to decrease duration of surgery and to tailor postoperative patient care according to risk predicted.

In conclusion, ACE-27 grade and ASA class are reliable predictors of major complications but ASA class had more impact on complications than ACE-27 grade. Duration of anaesthesia, preoperative radiotherapy and preoperative chemotherapy were also significant independent predictors of morbidity.
